# Can a kiss conquer all? The predictive utility of idealized first kiss beliefs on reports of romantic love among U.S. adults

**DOI:** 10.3389/fpsyg.2023.1256423

**Published:** 2023-12-07

**Authors:** Ashley E. Thompson, Madeleine R. Hill, Julia M. Record

**Affiliations:** Department of Psychology, University of Minnesota Duluth, Duluth, MN, United States

**Keywords:** idealized kissing beliefs, romantic love, romantic attachment, romantic kissing, romantic beliefs

## Abstract

Research indicates that idealized romantic expectations and the extent to which they are met, are important predictors of relationship outcomes (e.g., love). However, no studies have investigated the impact of idealized beliefs associated with specific behaviors (e.g., kissing) on reports of romantic love. Thus, the two studies comprising this research assessed the association between idealized beliefs related to one’s first romantic kiss with their current partner, unmet first kiss expectations, and reports of romantic love. Romantic attachment was also examined as a moderator. In Study One, the First Kiss Beliefs Scale was created and the results from 208 adults revealed that increased endorsement of idealized first kiss beliefs was associated with greater romantic love (*r* = 0.25). Romantic attachment also moderated this relationship, such that idealized first kiss beliefs significantly predicted love for those high in attachment anxiety and low in avoidance (*β* = 0.68 and *β* = 0.18, respectively). In Study Two, the First Kiss Beliefs Scale was modified to assess outcomes and expectations to capture unmet expectations. The results from 234 adults indicated that idealized first kiss beliefs predicted a greater proportion of the variance in romantic love (*sr^2^* = 0.10) than did unmet expectations (*sr^2^* = 0.07). A three-way interaction was also detected such that, among those low in attachment anxiety, the relationship between kissing beliefs and love was positive for those high in attachment avoidance and negative for those low. These results indicate that idealized first kiss expectations with one’s current romantic partner are important predictors of love (beyond whether these expectations were met), particularly for those high in attachment insecurity. Implications are discussed for practitioners and those in the primary stages of romantic relationships.

## Introduction

Romantic love has been conceptualized as having a lasting duration (i.e., commitment), an intense desire for physical and emotional union, as well as empathy and concern for a partner’s well-being (Gottschall and Nordlund, 2006). Additionally, in Sternberg’s (1986) groundbreaking work, romantic love is described as the interplay of intimacy, commitment, and passion. Furthermore, romantic love is characterized by a range of cognitive, affective, behavioral, social, and physiological activity (e.g., Aron et al., 2005; Acevedo et al., 2012; Fletcher et al., 2015; Sternberg and Sternberg, 2018). Researchers have argued that romantic love serves a variety of functions related to mate selection/pair-bonding and also as a prerequisite for relationship longevity and satisfaction (Dion and Dion, 1996; Willi, 1997). As a result, romantic love has been associated with greater feelings of self-fulfillment, self-expression (Dion and Dion, 1991), self-esteem, subjective well-being (Acevedo and Aron, 2009), and relationship satisfaction (e.g., Hendrick et al., 1988; Morrow et al., 1995; Vedes et al., 2016; Moore and Campbell, 2020).

### Idealized romantic beliefs and relationship outcomes

Although romantic love is a near-universal phenomenon (e.g., Buss, 2019; Sorokowski et al., 2021), empirical studies indicate that it manifests differently cross-culturally (Karandashev, 2015), particularly beliefs regarding what constitutes love in an ideal romantic relationship (i.e., romantic beliefs; Sprecher and Metts, 1989, 1999). For example, some research indicates that individuals from more individualistic cultures more commonly endorse overidealized romantic beliefs (akin to fairy tales), whereas individuals from collectivistic cultures tend to perceive passionate overidealized love as an illusory and expect it to come to an end as more “realistic/ enduring” love sets in (e.g., de Munck et al., 2011).

Although these romantic beliefs pertaining to love have been investigated for decades (e.g., Hobart, 1958) pioneers in the field, Sprecher and Metts (1989), were the first to comprehensively conceptualize and assess these beliefs among individuals in Western cultures. In fact, in 1989, Sprecher and Meets developed and validated the Romantic Beliefs Scale, which included items derived from several pre-existing romanticism scales. The resulting scale revealed that romantic beliefs were comprised of several components including resiliency amidst relationship obstacles, beliefs that there is only one true love, and that love can be accomplished at first sight.

From this work, the romantic belief ideology has been used to understand relationship, courtship, and romance scripts (i.e., cognitive structures that contain information relating to the key events that take place in romantic relationships; Ginsburg, 1988). In fact, many relationship scripts include elements related to “love at first sight,” “love can conquer all” and/or “love is blind,” all of which are commonly held romantic beliefs. It is posited that these scripts serve as a tool to guide behavior, particularly in times of uncertainty (Rose and Frieze, 1993). Thus, research reveals that relationship scripts predict one’s own thoughts and behaviors as well as those of their romantic partner(s) (Sprecher and Metts, 1989, 1999; Driesmans et al., 2016).

Consequently, scholars using the romantic belief framework and the relationship script framework have determined that these scripts (commonly containing over-romanticized beliefs) contribute to various relationship outcomes. Specifically, endorsing idealized romantic beliefs to a greater extent has been associated with overlooking a partner’s negative qualities (Murray and Holmes, 1997; Karandashev, 2019), maintaining the relationship for a longer duration (Ogolsky et al., 2017), seeing less decline in marital satisfaction over time (Murray et al., 2011), and reporting greater relationship satisfaction and commitment (Vannier and O’Sullivan, 2017). Additionally, Sprecher and Metts (1999) found that participants who reported more romantic love for their partner also endorsed idealized romantic beliefs to a greater extent.

### Idealized romantic kissing beliefs

Although various studies have examined the endorsement of idealized romantic beliefs (e.g., Vannier and O’Sullivan, 2017), no research has explored idealized beliefs toward specific intimate/romantic behaviors, such as romantic kissing. Romantic kissing (defined as “lip-to-lip contact that may or may not be prolonged between two individuals in a sexual, intimate setting;” Thompson et al., 2017, p. 1) is often the first sexual behavior that an individual engages in, with many individuals having their first romantic kiss before graduating high school (Regan et al., 2004). Additionally, romantic kissing is the most frequently engaged in sexual behavior (Welsh et al., 2005) with most romantic couples reporting kissing at least once each day (Busby et al., 2022). Thus, resulting from the high frequency of romantic kissing (Welsh et al., 2005) as well as Sprecher and Metts’ (1999) findings that idealized romantic belief endorsement positively predicted romantic love, it is reasonable to expect that idealized beliefs related to one’s first romantic kiss with their current romantic partner would increase reports of romantic love toward that partner.

Evolutionary psychologists argue that kissing plays an important role in successful reproduction, as kissing can provide insight into whether a potential partner is genetically fit for reproduction (Wlodarski and Dunbar, 2014). As partners kiss, olfactory cues (e.g., partner’s scent) provide insight into a partner’s health (Durham et al., 1993) and reproductive status (Fullagar, 2003; Wlodarski and Dunbar, 2013). Furthermore, romantic kissing plays a role in love and commitment such that kissing during a sexual experience is associated with sexual satisfaction and orgasm consistency (Busby et al., 2022), whereas kissing frequency has been associated with relationship and sexual satisfaction (Welsh et al., 2005; Wlodarski and Dunbar, 2013).

In addition to romantic kissing serving as a mate selection tool (e.g., Wlodarski and Dunbar, 2014), it has been argued that a first kiss can serve as a catalyst for romantic relationship initiation and solidification. Specifically, one study conducted by Wlodarski and Dunbar (2013) found that participants overall reported that a first romantic kiss has altered their feelings of romantic attraction toward a partner. Moreover, participants who more highly rated their partners as “good” kissers reported higher sexual frequency and relationship satisfaction than participants who provided lower ratings. Taken together, it is possible that first kisses that meet or exceed expectations (i.e., the partner was a “good” kisser), result in higher-quality relationships. However, despite the influential role of a first kiss experience on romantic attraction, the impact of idealized first kiss expectations on other areas of a relationship functioning, such as romantic love for one’s partner, has yet to be assessed. Thus, the current research developed a novel measure of idealized first romantic kiss beliefs and used this measure to assess whether these beliefs predicted reports of romantic love for one’s current romantic partner.

### The role of romantic attachment

Given that there is no existing literature regarding the impact of idealized first kiss beliefs on romantic love, the role of romantic attachment has yet to be explored. Romantic attachment was derived from Attachment Theory, which was first proposed by Bowlby (1958) to explain the emotional bond in a caregiver-child relationship and has since been extended to the study and understanding of romantic relationships (Hazan and Shaver, 1987; Fraley and Shaver, 2000; Simpson and Rholes, 2017). Attachment Theory posits that the physical proximity and attentiveness of a childhood attachment figure will result in the formation of a subsequent attachment style (e.g., secure, insecure-avoidant, or insecure-anxious; Bowlby, 1958, 1969; Ainsworth et al., 1978).

Romantic attachment was first conceptualized by Hazan and Shaver (1987), which deemed that attachment styles are relatively stable across the lifespan, in which affectional bonds with romantic partners are formed in similar ways to those between infants and their caregivers. These attachment styles have been proposed to differ according to how romantic love is experienced, establishing two broad dimensions: secure and insecure. There are two types of insecure attachment styles: anxious and avoidant (in which individuals can be high or low in one or both dimensions). First, those scoring high on anxious attachment tend to report relatively high levels of negative emotion, feel dependent on romantic partners, and fear abandonment. They have also been shown to experience romantic love through their tendency to overestimate threats within their relationships more than individuals scoring low in anxious attachment (Hazan and Shaver, 1987; Brewer and Forrest-Redfern, 2022). Second, those scoring high on avoidant attachment often display low levels of emotionality and experience romantic love through self-reliance to a greater extent than individuals scoring low in avoidant attachment (Sanford, 1997). On the contrary, those scoring low on both attachment avoidance and anxiety are referred to as “secure” and have regularly been found to experience romantic love through more happiness, trust, and friendship (Hazan and Shaver, 1987).

Research on relationship outcomes had indicated that those insecurely attached (i.e., scoring high on attachment anxiety and/or avoidance) report lower relationship satisfaction than do those scoring high in attachment security (e.g., Candel and Turliuc, 2019; Vollmann et al., 2019; Londero-Santos et al., 2020). Thus, researchers have investigated the extent to which those adopting insecure attachment styles adopt idealized romantic beliefs (e.g., Feeney and Noller, 1991; Hart et al., 2012, 2013; Jin and Kim, 2015). The results of these studies found that higher scores in attachment anxiety were positively associated with idealized romantic belief endorsement, whereas higher scores in attachment avoidance were negatively associated with idealized romantic belief endorsement. Furthermore, in a qualitative study conducted by Feeney and Noller (1991), participants gave verbal descriptions of their current romantic partners. Within these descriptions, romantic attachment was assessed via the coding of spontaneous references to attachment-related issues (e.g., commitment) and a one-item measure from Hazan and Shaver (1987). Their results revealed that those high in attachment anxiety scored the highest in idealized romantic beliefs, whereas those high in attachment avoidance scored the lowest in idealized beliefs.

Thus, because of the association between romantic attachment (particularly anxious attachment) and idealized romantic beliefs, it is also possible that romantic attachment is associated with idealized first kiss beliefs. This body of research reveals the possibility that those higher in attachment anxiety would endorse idealized first kiss beliefs to a greater extent, subsequently increasing their reported romantic love for their current partner. Conversely, those higher in attachment avoidance would endorse idealized first kiss beliefs to a lesser extent, subsequently decreasing their reported romantic love for their current partner. Furthermore, because romantic love has been operationalized as a multidimensional attachment process (Hazan and Shaver, 1987), the current program of research examined the relationship between idealized first kiss beliefs, romantic attachment, and reports of romantic love for one’s current partner.

## The current research

In sum, this program of research was designed to (1) develop a novel scale assessing idealized first romantic kissing beliefs, (2) examine the relationship between idealized first romantic kiss beliefs and romantic love, and (3) to assess the impact of romantic attachment on the endorsement of idealized first romantic kiss beliefs and romantic love. Because of the well-documented associations between romantic beliefs, romantic attachment, and relationship outcomes, the moderating role of romantic attachment in the relationship between idealized first kiss beliefs and romantic love was also investigated for exploratory purposes. Based on the romantic belief theoretical framework (Sprecher and Metts, 1989), existing literature, and Attachment Theory (Hazan and Shaver, 1987; Fraley and Shaver, 2000), the following hypotheses were generated:

*H1*: Adults who endorse idealized first romantic kiss beliefs to a greater extent were expected to report greater romantic love for their current partner as compared to those who endorsed idealized first romantic kiss beliefs to a lesser extent.

*H2*: Adults who scored higher on anxious attachment were expected to endorse idealized beliefs to a greater extent, whereas adults who scored higher on avoidant attachment were expected to endorse idealized beliefs to a lesser extent.

## Study One

The purpose of Study One was to develop a scale assessing idealized first romantic kissing beliefs and to assess the extent to which scores on this scale were associated with romantic love and romantic attachment (H1 & H2).

### Method

#### Participants

A total of 300 U.S. adults were recruited from Amazon’s Mechanical Turk (MTurk). However, 48 were removed due to failing to complete the survey in its entirety and an additional 31 were omitted because of incorrect responses to attention check items. Finally, 13 participants were removed due to not being in a romantic relationship (11 single, 1 divorced, 1 widowed). Thus, the final sample was comprised of 208 participants (134 men, 73 women, and 1 “prefer not to disclose”). Participants reported a mean age of 35.28 (*SD* = 10.24) and an average relationship length of 57.02 months (*SD* = 84.41), or roughly 4.75 years. A total of 67.3% of participants were married, 14.4% were dating, 10.1% were in a monogamous relationship, 5.3% were in an open relationship, 2.4% were cohabiting, and 0.5% were in a polyamorous relationship. The majority of participants identified as White (61.1%), followed by Asian (30.8%), African American (4.3%), American Indian or Alaska Native (1.9%), and lastly multiple races (1.4%). In addition, many identified as heterosexual (80.3%), followed by bisexual (16.8%), gay (1.9%), and pansexual (0.5%). On average adults in Study One reported a mean relationship length of 51.86 months (*SD* = 69.55), or just over 4 years.

#### Measures

##### First Kiss Beliefs Scale

The First Kiss Belief Scale (FKBS) was developed for the purposes of Study One. In doing so, undergraduate and graduate research assistants were responsible for developing a list of items that captured idealistic beliefs related to one’s first romantic kiss with their current romantic partner. After doing so, an initial list of 21 items were piloted using a sample of 20 undergraduate students in which difficult-to-comprehend items or those that did not fit were removed. Finally, pilot participants were asked to generate items that may have been missing. In sum, nine items were removed and two were added to the initial list.

The final draft of the FKBS included 14 items, all of which assessed the extent to which participants endorsed idealized kissing beliefs via a 7-point Likert scale, ranging from (1) not at all, to (7) very much. Participants received the following instructions “below are a series of questions asking about your expectations related to your first romantic kiss with your current romantic partner. When responding to each item, please reflect on your first romantic kiss with your partner (defined as lip-to-lip contact with someone of a sexual or romantic nature). If you have more than one romantic partner, please reflect on the partner you spend the most time with.” Sample items consisted of “to what extent should your first kiss turn you on?” and “to what extent should your first kiss give you ‘butterflies’?” with higher scores reflecting a greater endorsement of idealized kissing beliefs.

##### Experiences in Close Relationships Scale (ECR Scale)

The ECR Scale (Brennan et al., 1998) is a 36-item scale (divided into two subscales) that assessed insecure (anxious and avoidant) romantic attachment. The ECR Avoidance subscale contained 18 items that assessed discomfort with closeness (e.g., “I try to avoid getting too close to my partner”), whereas the ECR Anxiety subscale contained 18 items that assessed concern with abandonment (e.g., “I worry that my romantic partner will not care about me as much as I care about them”). Responses were assessed using a 7-point Likert scale ranging from 1 (strongly disagree) to 7 (strongly agree), with higher scores reflecting greater insecure attachment. Both anxiety and avoidance subscales demonstrated adequate discriminant validity (*r* = 0.17; Wei et al., 2007, p. 191), test–retest reliability (0.70; Wei et al., 2007), and internal consistency (Anxiety: *α* = 0.91, Avoidance: *α* = 0.94; Brennan et al., 1998). In Study One, the Avoidance (*α* = 0.85) and Anxiety subscales (*α* = 0.96) both demonstrated great internal consistency.

##### Demographics questionnaire

Participants provided information about their race/ethnicity, gender, age, sexual identity, relationship status, relationship length, and kissing history. They were also required to report on the extent to which they loved their partner via a 4-point response scale, ranging from 1 (not at all) to 4 (a lot).

#### Procedure

Upon Institutional Review Board (IRB) approval, participants were recruited to complete this study from a recruitment message on MTurk. Eligible participants (at least 18 years of age, English-speaking, had experience with romantic kissing, and currently in a relationship) were given an electronic consent form that outlined further details of the study (e.g., estimated time of completion, compensation information, IRB/PI contact information). Participants were then instructed to complete the FKBS and the ECR Scale, followed by a series of demographic questionnaires (in that order). Upon completion, participants were given an electronic debriefing form and were thanked for their participation. The study took 20 min to complete, and participants were compensated $2.00 USD into their MTurk accounts.

#### Data cleaning and preparation

Using the 10 participants-per-item guideline (Everitt, 1975), the sample size was considered adequate for performing an exploratory factor analysis. Approximately 3.7% of data was missing at the participant level and missing values were treated using mean substitution via the factor analysis command in SPSS. Although no outliers were identified on any of the FKBS items, the majority of items did demonstrate significant skew and the results should be interpreted with caution. Following initial data cleaning, a maximum likelihood exploratory factor analysis was computed with a promax rotation. The results produced from the scree plot and parallel analysis revealed that a single-factor solution was best and accounted for 52.39% of the variance. To determine which items to retain, factor loadings were reviewed. No items failed to load at 0.50 or higher, thus all 14 items were retained (see Table 1 for descriptive statistics for all items). To assess the internal consistency of the FKBS, Cronbach’s alpha was calculated. The results revealed that the FKBS had excellent scale reliability (*α* = 0.93).

**Table 1:** Means and standard deviations for the items in the First Kiss Beliefs Scale.

FKBS scale items	*M* (SD)
To what extent do you believe your first kiss should be a memorable event?	5.65 (1.34)
To what extent should you feel a lot of chemistry in your first kiss?	5.58 (1.29)
To what extent should your first kiss give you “butterflies?”	5.55 (1.48)
To what extent do you believe your first kiss is very important?	5.53 (1.44)
To what extent should your first kiss turn you on?	5.52 (1.30)
To what extent do you believe your first kiss needs to be exciting?	5.50 (1.32)
To what extent do you believe your first kiss is a really big deal?	5.48 (1.32)
To what extent should you feel “fireworks” from your first kiss?	5.46 (1.47)
To what extent does your first kiss need to have a spark?	5.43 (1.39)
To what extent do you believe your first kiss needs to be magical?	5.29 (1.51)
To what extent should your first kiss leave you speechless?	5.27 (1.52)
To what extent should your first kiss take your breath away?	5.25 (1.47)
To what extent do you believe you should feel electricity from your first kiss?	5.22 (1.46)
To what extent should you feel the whole world blur around you during your first kiss?	5.08 (1.58)

*N* = 208.

After finalizing the FKBS, outliers and skew were assessed for all scales and items of interest. Although no outliers were identified, the two subscales on the ECR Scale demonstrated significant skew (computed by dividing the skew statistic by the skew standard error). The skew on these variables was resolved via a square root and a logarithmic transformation. It is worth noting that all descriptive statistics are reported below in raw values.

To ensure sufficient power to conduct the exploratory moderation model, a sensitivity analysis using G*Power 3.1 (Faul et al., 2009) was conducted. The results revealed that the moderation analysis was sufficiently powered (80%) to detect a small-to-medium effect (*f^2^* = 0.05; *F* = 2.65) with an *p* value of = 0.05. Finally, exploratory analyses were conducted to assess the relationships between all primary variables and some demographic items (e.g., age, gender, relationship length). The results revealed that the demographic variables were not significantly correlated with idealized kissing beliefs or reports of romantic love (*p*s > 0.05).

### Results

#### Descriptive results

Preliminary descriptive analyses revealed that people reported a mean FKBS score of 5.39 (*SD* = 1.05) which indicates that participants reported fairly idealized or over-romanticized beliefs pertaining to their first romantic kiss with their current partner. Scores on the ECR Scale suggest that the sample endorsed avoidance items to a greater extent than anxious items, with a mean score of 4.75 (*SD* = 0.93) on the Avoidance subscale and 3.92 (*SD* = 1.57) on the Anxiety subscale. Finally, scores on the items assessing the extent to which participants “loved their partner” revealed that nearly everyone in the sample was at least somewhat in love with their current romantic partner, as can be seen by a mean of 3.35 (*SD* = 0.81) on a 4-point scale. In fact, 116 participants (52.5%) reported a value of 4 or that they loved their partner “a lot.”

#### Correlational results

To assess H1 and H2, Pearson-product moment correlation coefficients were computed using the scores on the FKBS, the love item, and the two subscales of the ECR Scale (see Table 2). These results support our H1, that those scoring higher on the FKBS reported being in love with their current partner to a greater extent than those scoring lower. In addition, H2 was partially supported such that those high in both anxious and avoidant attachment scored higher on the FKBS than did those scoring lower. Meanwhile, these results contrast with our prediction that those scoring high in avoidant attachment would yield lower FKBS scores. To explore whether romantic attachment moderated this relationship, a moderated moderation analysis was conducted using Andrew Hayes’ PROCESS macro (Model 3; Hayes, 2013). In the analysis, FKBS scores were entered as the predictor variable, scores on the love item as the outcome variable, and ECR subscale scores as the moderators.

**Table 2:** Correlation coefficients for the FKBS, love item, and ECR subscales for Study One.

Study variables	Pearson-product moment correlation coefficients			
	FKBS scores	Love scores	ECR-anxiety scores	ECR-avoidance scores
FKBS scores	--	--	--	--
Love item scores	0.25***	--	--	--
ECR-anxiety scores	0.21**	−0.17*	--	--
ECR-avoidance scores	0.32***	0.15*	0.65***	--

*N* = 208. *p < 0.05, **p < 0.01, ***p < 0.001.

**Figure 1:**
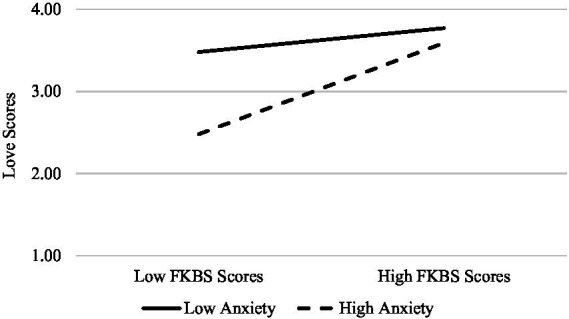
The relationship between FKBS scores and Love for those high and low in anxious attachment for Study One. Low anxiety = *M* – 1SD. High anxiety = *M* + 1SD.

The results revealed that (in addition to a significant association between anxious attachment and love; *β* = −0.43, *p* = 0.002) the interaction between FKBS scores and anxiety scores accounted for a significant amount of the variance in scores on the love item (*β* = 0.15, *p* < 0.001). To probe the interaction term further, a simple slopes analysis was conducted by examining the nature of the relationship between FKBS and romantic love scores separately for those high and low in anxious attachment. The results indicated that the relationship between idealized first kiss beliefs and romantic love was significantly stronger for those high in anxious attachment (*β* = 0.68, *p* < 0.001) than it was for those low in anxious attachment (*β* = 0.18, *p* = 0.02). The interaction between FKBS scores and avoidance scores also accounted for a significant amount of the variance in scores on the love item (*β* = −0.21, *p* = 0.01). To probe the interaction term further, a simple slopes analysis was conducted by examining the nature of the relationship between FKBS and romantic love scores separately for those high and low in avoidant attachment. The results of a second simple slopes analysis indicated that, the relationship between idealized first kiss beliefs and romantic love was significant for those low in avoidant attachment (*β* = 0.30, *p* < 0.001) but not for those high (*β* = 0.01, *p* = 0.91). See Figure 1 for a visual depiction. It is worth noting that the interaction between anxious and avoidant attachment (*β* = 0.09, *p* = 0.25) nor the three-way interaction (*β* = −0.11, *p* = 0.09) were statistically significant.

### Discussion

Given that idealized first kiss beliefs had yet to be assessed prior to this study, the first objective was to develop a scale measuring idealized first kiss beliefs. As expected, participants did endorse idealized first kiss beliefs to a high extent, which is indicated by a mean score of 5.39 on a scale from 1 to 7. This finding is supported by and extends past literature, which has revealed that individuals also commonly endorse idealized romantic beliefs to a high extent (e.g., Vannier and O’Sullivan, 2017). Proponents of the Romantic Beliefs Scale’s reliability and validity could potentially argue that idealized first kiss beliefs were a previously unknown, but salient subtype of idealized romantic beliefs, given the high endorsement of items that entail feelings of love arising from a first kiss (e.g., “To what extent should you feel a lot of chemistry in your first kiss.”). In particular, proponents may suggest that the FKBS could serve as an extension to the “love at first sight” dimension of the Romantic Beliefs Scale (i.e., love at first kiss).

Moreover, consistent with H1, the results from Study One indicated that those who endorsed idealized first kiss beliefs to a greater extent also reported being more in love with their current romantic partner than those endorsing these beliefs to a lesser extent. This relationship aligns and extends existing literature that has identified an association between idealized romantic belief endorsement and higher relationship satisfaction (Vannier and O’Sullivan, 2017; Kretz, 2019), which has been positively associated with love (Hendrick et al., 1988; Morrow et al., 1995; Vedes et al., 2016; Moore and Campbell, 2020).

As expected (H2), higher scores in attachment anxiety predicted endorsement of idealized first kiss beliefs to a greater extent. This finding is consistent with existing literature that states that those high in attachment anxiety are most likely to endorse idealized romantic beliefs (e.g., Feeney and Noller, 1991; Hart et al., 2012, 2013). Contrary to H2, however, higher scores in attachment avoidance predicted heightened endorsement of idealized first kiss beliefs, rather than a decreased endorsement. Although these results are surprising, they align with work conducted by Dinkha et al. (2015) indicating a positive association between attachment avoidance and parasocial relationships (defined as a one-sided relationship that an audience member fashions with a television personality). In particular, adults scoring high in attachment avoidance tend to form relationships with media characters in an effort to circumvent feelings of emotional closeness with their current partners. Additionally, individuals reporting more parasocial relationships tend to endorse romantic beliefs more strongly than those reporting fewer parasocial relationships (Jin and Kim, 2015). Thus, because those high in attachment avoidance are more inclined to participate in parasocial relationships and these relationships result in the endorsement of more overromanticized beliefs, the same is likely true for the endorsement of idealized first kiss beliefs.

For exploratory purposes, the moderating role of romantic attachment was assessed with regard to the association between idealized first kiss beliefs and reports of romantic love. The results revealed that the relationship between idealized first kiss beliefs and romantic love was stronger for those high in attachment anxiety as compared to low. It is possible that the endorsement of idealized beliefs could compensate for the overestimation of relationship threat and underestimation of the partner’s commitment (Brewer and Forrest-Redfern, 2022) that those with high attachment anxiety experience. Results also found that the relationship between idealized kissing beliefs and romantic love was stronger for those lower in attachment avoidance as compared to high. Since those high in attachment avoidance conceptualize intimacy as threatening and their partners as more undependable (Hazan and Shaver, 1987; Hart et al., 2013), a stronger relationship between idealized kissing beliefs and romantic love could be justified for those low in attachment avoidance as compared to high. In particular, those high in attachment avoidance could place less emphasis on the value of a first romantic kiss in an effort to distance themselves from the potential intimacy that could result.

Although Study One helped to progress literature on romantic love and idealized romantic beliefs, some limitations should be noted. First, a one item-measure was used to assess romantic love, which could have led to questionable reliability and validity (Diamantopoulos et al., 2012). Thus, Study Two incorporated a multi-item scale to assess love, as multi-item scales show stronger predictive validity than single-item scales.

Second, we do not know the extent to which unmet first kiss expectations predict romantic love. In fact, it is possible that hyper-romanticized beliefs contribute to more unrealistic romantic expectations (Spaulding, 1970; Glenn, 1991; Galician, 2004), resulting in relationships that fail to meet expectations and inevitably poor relationship outcomes (Vannier and O’Sullivan, 2017, 2018). Research reveals that unmet expectations have been associated with lower levels of sexual satisfaction, as well as higher levels of sexual distress and relationship conflict (Rosen et al., 2022). Additionally, unmet expectations have been identified as better predictors of decreased relationship satisfaction and commitment as compared to idealized romantic beliefs alone (Vannier and O’Sullivan, 2017). Thus, research is needed to explore the impact of unmet kissing beliefs on reports of romantic love.

## Study Two

To address limitations associated with the previous study, Study Two was designed to explore the extent to which unmet first kiss expectations predicted reports of romantic love (using a validated multi-item measure) in comparison to idealized first kiss beliefs. With this in mind, the following novel hypothesis was generated.

*H3*: Unmet first kiss expectations were expected to predict a greater proportion of the variance in reports of romantic love in comparison to idealized first kiss beliefs.

### Method

#### Participants

A total of 250 participants were recruited through Prolific ®. However, 10 were removed due to responding incorrectly to any of the attention check items, four due to duplicate IP addresses, and one more for not meeting the eligibility criteria. Thus, the sample was composed of 235 U.S. adults. Participants reported an average age of 39.49 years (*SD* = 12.37). A majority of participants identify as men (50.20%) followed by women (48.09%). In addition, 78.30% of participants reported being White, followed by Asian (9.79%) and Black or African American (8.94%). In total, 83% of participants reported identifying as heterosexual followed by bisexual (9.4%), gay (4.3%), pansexual (3.4%), lesbian (1.3%), queer (0.9%), asexual (0.4%), and 0.4% reported not knowing their sexual identity. Additionally, 59.6% of participants were married, 19.6% were in a monogamous relationship, 14.9% were cohabitating, 4.7% were in dating relationships, and 1.3% indicated a relationship status other than the previous. On average, adults in Study One reported a mean relationship length of 171.55 months (*SD* = 132.08), or approximately 14 years. Participants’ average age of their first romantic kiss with their current partner was 25.22 years (*SD* = 8.52).

#### Measures

##### First Kiss Beliefs Scale

The First Kiss Beliefs Scale (FKBS) was used to assess first kiss expectations in Study Two. The results of a second maximum likelihood EFA confirmed that a single-factor structure best portrayed the data (accounting for 64.78% of the variance). All items loaded at 0.65 or higher on the factor and items in the FKBS demonstrated great internal consistency, as evidenced by Cronbach’s alpha of 0.96.

##### First Kiss Outcome Scale (FKOS)

The First Kiss Outcome Scale (FKOS) was developed for the purpose of Study Two by revising the FKBS to assess the extent to which their first kiss met their expectations. Similar to the FKBS, it was composed of 14 items all rated on a 7-point Likert scale, ranging from (1) not at all to (7) very much. Sample items include “To what extent did you feel the whole world blur around you during your first kiss?” and “To what extent was your first kiss magical?”

Another maximum likelihood EFA was conducted to explore the factor structure of the FKOS. The results of a parallel analysis and visually inspecting the scree plot indicated that only one factor was needed to best summarize the data (accounting for 68.38% of the variance). All items loaded at 0.58 or higher and the FKOS proved to be internally consistent (*α* = 0.96).

##### Experiences in Close Relationships Scale (ECR Scale)

The ECR Scale (Brennan et al., 1998) was once again used in Study Two, with both scales demonstrating adequate internal consistency, Avoidance (*α* = 0.82) and Anxiety (*α* = 0.94).

##### Romantic Love Scale (RLS)

Romantic love (Rubin, 1970) was measured using the Romantic Love Scale (RLS), which is composed of 13 items on a 9-point Likert scale, ranging from (1) not at all true to (9) definitely true. Participants were instructed to think about their romantic partner while completing the measure. Sample items include “I find it easy to ignore my partner’s faults.” and “I would do almost anything for my partner.” The items in the RLS have a Cronbach’s alpha of 0.89, indicating the items to be internally consistent.

##### Demographics questionnaire

Similar to Study One, participants provided information about their race/ethnicity, gender, age, sexual identity, relationship status, relationship length, and kissing history.

#### Procedure

Upon Institutional Review Board (IRB) approval, participants were recruited for this online study through a recruitment message on Prolific®. Eligible participants (at least 18 years of age, from the United States, and in a current romantic relationship) were given an electronic consent form that further outlined specific study details. Participants then completed the FKBS, the Kissing Outcome Scale, the Romantic Love Scale, and a demographics questionnaire (in that order). Following study completion, participants were given an electronic debriefing form and thanked for their time. The study took approximately 10 min to complete, and participants were compensated with a $2.00 USD deposit to their Prolific accounts.

#### Data cleaning and preparation

To ensure sufficient power to conduct the exploratory moderation model, a sensitivity analysis was conducted for Study Two. The results revealed that the moderation analysis was sufficiently powered (80%) to detect a small-to-medium effect (*f^2^* = 0.04; *F* = 2.64) with a *p* value of = 0.05. Approximately 0.9% of data was missing at the participant level, thus missing values were dealt with using listwise deletion. Although there was only one outlier on the Anxiety subscale of the ECR Scale, the Avoidance subscale of the ECR Scale, and the FKBS, all outlier values were reported by the same participant. Thus, this individual was removed from all analyses, resulting in a final sample size of 234 participants. After reviewing the distributions for the variables of interest, the RLS and the Anxious subscale of the ECR Scale demonstrated significant skew. That said, the skew was resolved for both scales using a logarithmic transformation. Once again, all descriptive statistics are reported below in raw values.

In order to assess the extent to which participants idealized first kiss beliefs were unmet, difference scores (i.e., D_kiss_ scores) were computed by subtracting FKOS scores from FKBS scores. Consequently, negative D_kiss_ scores indicate unmet expectations, positive D_kiss_ scores indicate exceeded expectations, and D_kiss_ scores approaching 0 suggest one’s first kiss expectations were met. Finally, age, gender, and relationship length were not significantly correlated with idealized kissing beliefs or reports of romantic love (*p*s > 0.05).

### Results

#### Descriptive results

Consistent with Study One, descriptive analyses indicated a mean FKBS score of 5.03 (*SD* = 1.30), confirming that participants reported fairly over-romanticized first kiss beliefs. Again, the sample endorsed avoidance items to a greater extent than anxious items, with a mean score of 4.44 (*SD* = 0.57) on the Avoidance subscale and 2.79 (*SD* = 1.13) on the Anxiety subscale. With regard to our new measure of romantic love, a mean score of 7.05 (*SD* = 1.32) out of 9 suggested that participants were very in love with their current romantic partner. Scores on the FKOS revealed that participants’ expectations were likely met (even exceeded in some cases), as evidenced by a mean score of 5.07 (*SD* = 1.49). Finally, the mean D_kiss_ score was 0.03 (*SD* = 0.99), revealing that participants’ first kiss expectations were fairly consistent with their first kiss outcomes.

#### Correlational and predictive results

Pearson-product moment correlation coefficients were once again used to assess H1 and H2. Consistent with Study One, the results indicated that FKBS scores were positively associated with RLS scores. In addition, FKBS scores were positively associated with both the Avoidance and Anxiety subscales of the ECR Scale. Interestingly, D_kiss_ scores were positively associated with RLS scores, but to a lesser extent than FKBS scores (See Table 3).

**Table 3:** Correlation coefficients for the FKBS, D scores, RLS, and ECR subscales for Study Two.

Study variables	Pearson-product moment correlation coefficients				
	FKBS scores	D scores	RLS scores	ECR-anxiety scores	ECR-avoidance scores
FKBS scores	--	--	--	--	--
D scores	−0.18**	--	--	--	--
RLS scores	0.28***	0.0.22***	--	--	--
ECR-anxiety scores	0.16*	−0.05	−0.15*	--	--
ECR-avoidance scores	0.20**	0.08	0.57***	−0.17*	--

*N* = 234. *p < 0.05, **p < 0.01, ***p < 0.001.

To assess H3 (whether unmet expectations were a better predictor of romantic love than idealized first kiss beliefs) a hierarchical linear multiple regression was conducted with unmet expectations entered as the predictor variable in block one and idealized beliefs in block two. The results indicated that, in block one, the D_kiss_ scores predicted a significant amount of the variance is RLS scores, *R^2^* = 0.05, *F*(1, 232) = 11.38, *p* < 0.001. When the FKBS scores were entered on block two, they also predicted a significant amount of the variance in RLS scores, *R^2^* = 0.15, *F_change_*(1, 231) = 27.91, *p* < 0.001. In fact, contrary to our expectations (H3), an examination of the semi-partial correlations revealed that FKBS scores predicted a greater proportion of unique variance in RLS scores (*β* = 0.33, *sr^2^* = 0.10, *p* < 0.001) than did the D_kiss_ scores (*β* = 0.27, *sr^2^* = 0.07, *p* < 0.001).

**Figure 2:**
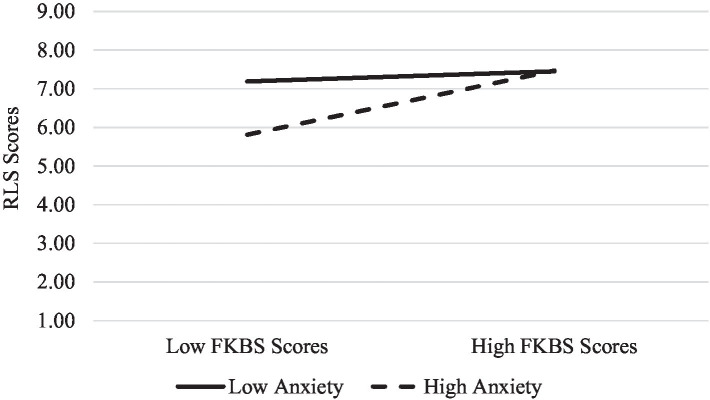
The relationship between FKBS scores and RLS for those high and low in anxious attachment for Study Two. Low anxiety = *M* – 1SD. High anxiety = *M* + 1SD.

**Figure 3:**
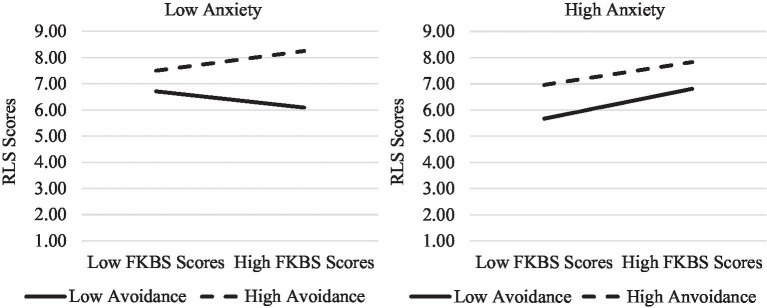
The three-way interaction between FKBS scores, ECR scale scores, and RLS scores for Study Two. Low anxiety/Avoidance = *M* – 1SD. High anxiety/Avoidance = *M* + 1SD.

Finally, to examine the moderating role of romantic attachment on the relationship between FKBS scores and RLS scores, another moderated moderation analysis was conducted. Again, FKBS scores were entered as the predictor variable, scores on the RLS as the outcome variable, and ECR subscale scores as the moderators. In addition to the significant associations between kissing beliefs (*β* = 0.20, *p* < 0.001), anxious attachment (*β* = −0.12, *p* = 0.04), avoidance attachment (*β* = −0.16, *p* = 0.001) and romantic love, the results revealed that the interaction between FKBS scores and anxiety scores accounted for a significant amount of the variance in RLS scores (*β* = 0.17, *p* = 0.004). To probe the interaction term further, a simple slopes analysis was conducted by examining the nature of the relationship between FKBS and RLS scores separately for those high and low in anxious attachment. The results indicated that the relationship between idealized first kiss beliefs and romantic love was significant for those high in anxious attachment (*β* = 0.63, *p* < 0.001) but not for those low in anxious attachment (*β* = 0.10, *p* = 0.21). See Figure 2 for a visual depiction. However, unlike Study One, the interaction between FKBS scores and avoidance scores did not account for a significant amount of the variance in RLS scores (*β* = 0.12, *p* = 0.23). Thus, no follow-up analyses were conducted. Although the interaction between anxious and avoidance attachment was not significant (*β* = −0.07, *p* = 0.20), the three-way interaction did account for a significant amount of the variance in RLS scores (*β* = −0.16, *p* = 0.001). The interaction between attachment avoidance and kissing beliefs varied among those high and low in attachment anxiety, such that attachment avoidance did not alter the relationship between kissing beliefs and romantic love for those high in attachment anxiety but it did for low. In particular, among those low in attachment anxiety, the relationship between kissing beliefs and love was positive for those high in attachment avoidance and negative for those low (see Figure 3).

### Discussion

Study Two expanded on Study One by incorporating a multi-item scale to assess romantic love, as well as assessing kissing outcomes to determine whether idealized first kiss beliefs or unmet expectations more strongly predicted reports of romantic love. Consistent with Study One, the results from Study Two indicated that individuals tend to strongly endorse idealized first kiss beliefs and that these beliefs predict romantic love. However, this relationship was once again moderated by anxious romantic attachment (but not avoidance), such that the association between idealized first kiss beliefs and romantic love was significant for those high in anxious attachment but not low. Overall, these results confirm that there are benefits to holding idealized beliefs regarding a first romantic kiss and that these benefits appear to be strongest for those anxiously attached.

The results from Study Two also indicated that both idealized first kiss belief endorsement and unmet expectations predicted romantic love. Contrary to H3, however, the predictive utility of idealized first kiss belief endorsement on reports of romantic love was greater than that of unmet expectations. Although there are numerous potential explanations for this finding, it may relate (in part) to optimism. In fact, research indicates that adults who report greater dispositional optimism report higher relationship quality as compared those who are less optimistic (Leahy et al., 2023). Thus, because those endorsing idealized first kiss beliefs to a greater extent are likely more optimistic about romantic relationships, they are more satisfied with their relationship and ultimately more in love. It is also possible that our findings relate to the degree to which expectations were met in the first place. For example, the majority of participants in Study Two reported that their first kiss expectations were met or even exceeded, whereas Vannier and O’Sullivan (2017) reported that expectations were unmet, on average. This difference in the extent to which expectations were met could have altered the extent to which idealized first kiss endorsement and unmet expectations predicted reports of romantic love.

Of note, the results from Study Two revealed that increased endorsement of idealized first kiss beliefs predicted more unmet expectations within a relationship. Although this finding contrasts with that from Vannier and O’Sullivan’s (2017) study, it can be supported by past literature suggesting that idealized romantic belief endorsement could aid in formulating unfeasible, and possibly unattainable romantic expectations (Spaulding, 1970; Glenn, 1991; Galician, 2004). Moreover, it is possible that reports of romantic love could have been highest for those who adopted high first kiss expectations, and these expectations were met or even exceeded.

Finally, the three-way interaction between kissing beliefs, anxious, and avoidant attachment revealed that the association between idealized kissing beliefs on reports of romantic love was positive for everyone except those low in both anxious and avoidant attachment (i.e., securely attached individuals). It is possible that those who are securely attached place less emphasis on romantic kissing beliefs when evaluating their relationship. In fact, research indicates that insecurely attached adults endorse more relationship-specific irrational beliefs (e.g., “people who love each other know exactly what each other’s thoughts are without a word even being said,” “I take it as a personal insult when my partner disagrees with an important idea of mine”) than those securely attached (Stackert and Bursik, 2003). Consequently, adults who are securely attached who resort to endorsing idealized kissing beliefs may be doing so in times of distress as a tool to overcome dissatisfaction or in an attempt to savor a dissolving relationship.

## General discussion

Despite the frequency of romantic kissing in Western cultures (Welsh et al., 2005), as well as the well-supported links between idealized romantic beliefs and relationship satisfaction (e.g., Vannier and O’Sullivan, 2017), the current program of research was the first to assess the beliefs that individuals hold when entering a first romantic kiss and the extent to which these beliefs predict romantic love. The first objective of this research was to assess idealized first kiss belief endorsement via the development of a novel measure. In creating this measure, we were able to determine that individuals do, in fact, endorse idealized first kiss beliefs. In fact, holding idealized first kiss beliefs was very commonplace among respondents. The pervasiveness of idealized first kiss beliefs among our sample could possibly be explained via Cultivation Theory (Gerbner and Gross, 1976; Gerbner et al., 1980; Lichter et al., 1994), in that the high prevalence (76% television shows, Timmermans and Van den Bulck, 2018) and the overidealized depiction of first romantic kisses within mainstream media (e.g., first kisses presume living “happily ever after” with one’s true love; Hefner et al., 2017; Dajches and Aubrey, 2020) leads viewers to adopt equally idealized notions about first romantic kisses in the real world.

Across both studies, H1 was supported. In particular, greater idealized first kiss belief endorsement predicted higher reports of romantic love (expect among those securely attached). This is intuitive given the aforementioned roles of kissing frequency (Welsh et al., 2005) and idealized romantic belief endorsement (Vannier and O’Sullivan, 2017; Kretz, 2019) in promoting relationship satisfaction. This is also consistent with the romantic belief framework. Because idealized kissing beliefs influence relationship/courtship scripts, kissing likely has a large role in predicting expectations in romantic relationships (Sprecher and Metts, 1989, 1999; Driesmans et al., 2016). In fact, our findings support existing literature documenting the importance of one’s first kiss (Robinson, 1992; Regan et al., 2007; Simpson et al., 2020). Evidence of the importance of kissing can be gleaned from research by Rice et al. (2017) indicating that people can remember approximately 90% of the details surrounding their first romantic kiss (more than the proportion of details remembered relating to one’s sexual debut).

Partially consistent across both studies was H2. In particular, higher attachment anxiety consistently predicted endorsement of idealized first kiss beliefs. It is no surprise that attachment anxiety was positively correlated with idealized first kiss beliefs because of the existing literature linking anxious attachment to idealized romantic beliefs (e.g., Feeney and Noller, 1991; Hart et al., 2012, 2013). However, additional research should be conducted to explore the relationships between attachment avoidance and idealized first kiss beliefs. In fact, it is possible that the ubiquitous negative association between attachment avoidance and idealized romantic belief endorsement documented in previous studies (e.g., Feeney and Noller, 1991; Hart et al., 2012, 2013; Jin and Kim, 2015) may not generalize to specific intimate behaviors such as one’s *first* romantic kiss.

Given the significant moderating role of romantic attachment on the relationship between idealized first kiss beliefs and reports of romantic love, adopting and endorsing idealized first kiss beliefs could be particularly useful for insecurely attached adults, particularly those high in attachment anxiety (as this was the only construct that consistently moderated the relationship across both studies). These findings could potentially be explained by the tendency for those scoring higher in attachment anxiety to seek reassurance to a greater extent (Clark et al., 2020), as well as report greater interpersonal attraction when given positive feedback (Sperling and Borgaro, 1995). Specifically, it is possible that idealized first kiss belief endorsement could have been used as a means of reassurance that their current partner loves them in return (i.e., positive feedback), which could have translated to increases in their own reports of romantic love. With regard to attachment avoidance, endorsing idealized first kiss beliefs may not be useful as people high in avoidance likely evade placing the same degree of emphasis on a first romantic kiss in an effort to reduce the threat of intimacy that may result. Nevertheless, more research exploring the impact of attachment avoidance on idealized first kiss beliefs is important in order to clarify the inconsistencies documented in the two current studies.

Finally, contrary to H3, idealized first kiss beliefs more strongly predicted reports of romantic love than did unmet expectations. Specifically, idealized first kiss belief endorsement explained two times as much of the variance (10%) in romantic love as compared to unmet expectations (5%). This supports research by Vannier and O’Sullivan (2017) that romantic beliefs (on their own predict relationship outcomes). Furthermore, our research suggests that entering a relationship with high first kiss expectations may be beneficial in promoting romantic love toward one’s current romantic partner, regardless of the potential for unmet expectations.

### Limitations and future directions

Although this program of research expanded our understanding of romantic kissing expectations (a severely understudied area), several limitations must be noted. First, all participants were asked to reflect on their first romantic kiss with their current romantic partner. Consequently, it is likely that our results were plagued by issues associated with recall bias considering that participants reported being in their current relationship for a substantial amount of time and likely were far past the courtship phase, particularly in Study Two (roughly 14 years). As a result, our participants may not have adequately remembered their expectations prior to their first romantic kiss. In fact, the recall bias often results in an overestimation in remembering past affect (Wirtz et al., 2003; Ben-Zeev et al., 2009; Colombo et al., 2019), such that people have a tendency to overestimate positively-valanced emotions. Consequently, it is plausible that people overestimated how much they idealized their first kiss because they are still with their current partner, whereas those no longer with their partner (who were not allowed to participate) likely would report different expectations. Researchers should work to replicate this research by recruiting individuals currently in the courtship phase of a relationship and following them longitudinally to assess their reports of romantic love. Additionally, researchers could recruit dyads to assess kissing beliefs and romantic love (allowing for comparisons for validity purposes) or, better yet, employ implicit measures to bypass issues with response biases.

Second, our study was comprised of U.S. adults who were currently in a romantic relationship. Thus, the results of our study likely fail to generalize to adults from other cultures. In fact, several studies have produced findings that counter the common Western belief that romantic partners express their desire for one another through romantic kissing (e.g., Jankowiak et al., 2015). Despite common depictions of romantic kissing in a variety of media, romantic kissing is only present in approximately 46% of cultures. Thus, kissing beliefs likely do not impact romantic love in many cultures the way it does in Western cultures.

Second, the scale we used to assess idealized first kiss expectations (FKBS) was novel and the validity still needs to be assessed. Thus, the extent to which this scale accurately and holistically assesses idealized first kiss beliefs remains unknown. Future studies should be used to validate the scale to ensure that all domains of idealized first kiss beliefs are accurately assessed. Relatedly, according to Classical True Score Theory (Gulliksen, 1987) the use of D_kiss_ scores in Study Two may yield concerns about the reliability of our results. In fact, statisticians have documented the problematic reliability of difference scores computed from highly correlated items/scales. Thus (although the scales themselves demonstrated excellent scale reliability), all results involving the D_kiss_ scores should be interpreted with caution.

Finally, the associations between idealized first kiss beliefs, romantic attachment, and reports of romantic love were strictly correlational. From this research program alone, we are unable to determine whether having high first kiss expectations increases reports of romantic love, or whether individuals report more romantic love for their current partners as a result of setting high expectations for their first kiss. We are also unable to determine whether scoring high in attachment anxiety and/or avoidance increases idealized first kiss belief endorsement. Thus, we encourage researchers to adopt innovative experimental designs to explore the causal relationship between idealized first kiss beliefs and romantic love, as well as the relationship between romantic attachment and idealized first kiss beliefs.

### Implications

In sum, the current research confirmed that adults do hold idealized first kiss beliefs and that these beliefs have important implications for romantic relationships, particularly the love reported for one’s romantic partner. Consequently, the results from our research have a variety of implications. First, the novel scale in our study demonstrated utility in understanding variations in romantic love. Thus, we encourage researchers to modify/expand the FKBS to assess other “firsts” in intimate behaviors (physical and/or emotional) other than kissing (e.g., sexual debut). In doing so, a more holistic understanding of how beliefs regarding novel behaviors impact romantic love and relationship functioning. Second, to support those in interpersonal distress and to promote romantic love between partners, items in the FKBS could serve as a guide for the beliefs individuals should endorse prior to engaging in first kisses with their current partners. Finally, these results could prove useful for clinicians and practitioners looking to improve the experience of romantic love. In fact, clinicians could encourage adults to internalize more idealistic kissing beliefs in an effort to promote and/or enhance feelings of romantic love.

## Data availability statement

The datasets presented in this study can be found in online repositories. The names of the repository/repositories and accession number(s) can be found at: https://osf.io/zjmby/?view_only=b3ab79df70ce45ac87cafffb468f69f6.

## Ethics statement

The studies involving humans were approved by University of Minnesota Institutional Review Board. The studies were conducted in accordance with the local legislation and institutional requirements. The ethics committee/institutional review board waived the requirement of written informed consent for participation from the participants or the participants’ legal guardians/next of kin because participants were recruited online via Prolific.

## Author contributions

AT: Conceptualization, Formal analysis, Investigation, Methodology, Project administration, Supervision, Validation, Writing – original draft, Writing – review & editing. MH: Writing – original draft, Writing – review & editing. JR: Writing – original draft, Writing – review & editing.
